# The diagnostic and prognostic significance of monitoring blood levels of immature neutrophils in patients with systemic inflammation

**DOI:** 10.1186/s13054-015-0778-z

**Published:** 2015-02-25

**Authors:** Tracey Anne Mare, David Floyd Treacher, Manu Shankar-Hari, Richard Beale, Sion Marc Lewis, David John Chambers, Kenneth Alun Brown

**Affiliations:** Intensive Care Unit, Guy’s and St Thomas’ NHS Foundation Trust, St Thomas’ Hospital, Westminster Bridge Road, London, SE1 7EH UK; Division of Asthma, Allergy and Lung Biology, Faculty of Life Sciences and Medicine, King’s College London, Great Maze Pond, London, SE1 9RT UK; Cardiac Surgical Research, The Rayne Institute (King’s College London), Guy’s and St Thomas’ NHS Foundation Trust, St Thomas’ Hospital, Westminster Bridge Road, London, SE1 7EH UK; Vascular Immunology Research Laboratory, Rayne Institute (King’s College London), St Thomas’ Hospital, Westminster Bridge Road, London, SE1 7EH UK

## Abstract

**Introduction:**

In this cohort study, we investigated whether monitoring blood levels of immature neutrophils (myelocytes, metamyelocytes and band cells) differentiated patients with sepsis from those with the non-infectious (N-I) systemic inflammatory response syndrome (SIRS). We also ascertained if the appearance of circulating immature neutrophils was related to adverse outcome.

**Methods:**

Blood samples were routinely taken from 136 critically ill patients within 48 hours of ICU entry and from 20 healthy control subjects. Clinical and laboratory staff were blinded to each other’s results, and patients were retrospectively characterised into those with SIRS (*n* = 122) and those without SIRS (*n* = 14). The patients with SIRS were further subdivided into categories of definite sepsis (*n* = 51), possible sepsis (n = 32) and N-I SIRS (*n* = 39). Two established criteria were used for monitoring immature white blood cells (WBCs): one where band cells >10% WBCs and the other where >10% of all forms of immature neutrophils were included but with a normal WBC count. Immature neutrophils in blood smears were identified according to nuclear morphology and cytoplasmic staining.

**Results:**

With the first criterion, band cells were present in most patients with SIRS (mean = 66%) when compared with no SIRS (mean = 29%; *P* <0.01) and with healthy subjects (0%). The prevalence of band cells was higher in definite sepsis (mean = 82%) than in patients with possible sepsis (mean = 63%; *P* <0.05) or with N-I SIRS (mean = 39%; *P* <0.001), and they had a sensitivity of 84% and a specificity of 71% for the detection of definite sepsis. With the second criterion (that is, patients with normal WBC counts), we noted that immature neutrophils did not differentiate any of the patient groups from one another. Patients who died within 1 week of blood sample provision had higher levels of myelocytes and metamyelocytes (median = 9%; *P* <0.05) than patients who died at 2 to 4 weeks (median =0.5%).

**Conclusions:**

Raised blood levels of band cells have diagnostic significance for sepsis, provided that measurements are not confined to patients with normal WBC counts, whereas an increased prevalence of myelocytes and metamyelocytes may have prognostic application.

## Introduction

The systemic inflammatory response syndrome (SIRS) identifies patients who are at high risk of developing organ failure. It is initiated by infections (sepsis) and by non-infectious (N-I) stimuli that include trauma, stress, cardiopulmonary bypass and pancreatitis [1]. In 1992, a consensus conference led by Bone and colleagues defined patients with SIRS as satisfying two of the four following conditions: temperature >38°C or <36°C, heart rate >90 beats/min, respiratory rate >20 breaths/min and a white blood cell (WBC) count >12 × 10^9^/L or <4 × 10^9^ L or >10% immature (band) forms of neutrophils in the circulation [2]. In a later conference, chaired by Levy *et al.*, these four criteria were included in the diagnostic criteria for sepsis as it was proposed that, although the SIRS concept was valid, it was considered to have limited diagnostic application [3]. Moreover, in this modified format, the identification of >10% immature neutrophils was now restricted to just a normal WBC count without reference to band cells. No explanation was provided for the exclusion of patients with abnormal WBC counts, and the omission of band cells may have been made in the belief that other immature neutrophils are contributing to this diagnostic criterion. To appreciate the latter consideration, it is necessary to recall the various forms of immature neutrophils that appear during granulopoiesis in the bone marrow. A 7-day mitotic stage (myeloblast → promyelocyte → myelocyte) is followed by a 7-day maturation stage (myelocyte → metamyelocyte → band cell → mature segmented neutrophil), whereupon mature neutrophils are held in storage pools before their entry into the circulation [4]. Factors controlling the release of neutrophils from the bone marrow are outlined in several informative reviews [5-8]. To date, most reports of circulating immature neutrophils in SIRS refer to band cells, the direct precursors of mature neutrophils, with limited reference to other myeloid progenitors, such as myelocytes and metamyelocytes [9-16].

It is of interest that one of the criteria for SIRS involves the appearance in the circulation of immature neutrophils, whose mature forms are essential for the elimination of foreign bacteria but whose untoward activities could lead to organ failure [17,18]. The appearance of band cells in the blood of patients with bacterial infections is commonly referred to as ‘a shift to the left’ [8]. Because SIRS is often initiated by infections, 90% of which are bacterial in origin [19], there is the question whether, in the intensive care unit (ICU) setting, elevated levels of circulating band cells are associated predominantly with N-I SIRS or with sepsis. We therefore investigated the relative merits of the WBC criteria of Bone *et al.* [2] and of Levy *et al.* [3] in identifying patients with SIRS and whether monitoring levels of all forms of immature neutrophils could help in differentiating patients with N-I SIRS from those with sepsis. It was recently shown in patients with septic shock that increased mortality was related to WBC counts [20] and that in patients with sepsis an increase in the number of immature neutrophils reflected disease severity and predicted patient deterioration [14,15]. On this basis, we also examined whether levels of immature neutrophils had a bearing on patient outcome.

## Material and methods

### Patients and controls

Blood samples, obtained from 136 consecutive patients within 48 hours of entry into the adult ICU, were examined by light microscopy for the distribution of immature neutrophils. Samples were acquired every weekday for 8 weeks. Later, the patients were retrospectively and independently categorised by two ICU consultants (MSH and DFT), who were unaware of the immature neutrophil data, into those with SIRS (*n* = 122) and those without SIRS (*n* = 14). Patients were defined as having SIRS if they satisfied at least two of the recognised criteria, but without reference to the number or distribution of WBCs. We undertook this omission because, in the Levy definition, >10% immature neutrophils were recorded in patients with a normal WBC count, and, if we had used a neutrophilia or a neutropenia as one of the features of SIRS, then a potentially important subgroup of patients (that is, those with normal WBC counts) would have been removed from the study. The patients with SIRS were further differentiated into three groups: (1) 51 patients with definite sepsis (either microbiological documentation of an infective organism or where both consultants had a very strong clinical suspicion of infection in the absence of positive microbiology), (2) 32 patients with possible sepsis (no relevant confirmatory microbiology, but where one of the consultants had a strong clinical suspicion) and (3) 39 patients with N-I SIRS (neither clear microbiological evidence nor any clinical suspicion of infection) [21].

In the retrospective analysis, the two reviewing consultants examined clinical data held in contemporaneous notes and collected prospectively from the Clinical Information System ((CIS) CareVue™; Philips Medical Systems, Eindhoven, the Netherlands) alongside radiological and microbiological findings. This CIS also included the clinical impressions of the consultant physician in charge of patient care on the day when the blood sample was collected, who was independent of the study team. There was 100% concordance between the reviewing consultants in defining definite sepsis and N-I SIRS. For the remaining patients, both consultants felt that, although antibiotic therapy was initiated and the differential diagnosis of clinical deterioration included sepsis, there were no microbiological data to support the confirmation of sepsis. Furthermore, there were other possible clinical reasons for deterioration of patients, and this group was thus labelled as having possible sepsis. For this group, there was nearly 90% concordance between the study consultants. Disagreements were resolved by joint care review by the study team blinded to the laboratory results of the prevalence of immature neutrophils. Of the 136 patients studied, 33 died within 30 days of ICU stay.

Also included in the investigation were blood samples from 20 normal healthy members of staff. Table 1 presents the patient demographics, and Table 2 describes the sites of infection and organisms identified by microbiological culture in patients with sepsis. Of the 51 patients with definite sepsis, 29 had community acquired infections and 22 had hospital acquired infections. Ethical approval for this non-interventional, observational study was not required, as all of the tests done were routinely performed for patient assessment in the ICU and no additional blood provision was required.

**Table 1:** **Patient details and source of infections** ^**a**^

	**Number of patients**	**Age (yr)**	**% Male**	**WBCs (×10** ^**9**^ **/L)**	**Platelets (×10** ^**9**^ **/L)**	**CRP (μg/ml)**
Definite sepsis	51	62 ± 16	59	14 ± 9	232 ± 118	173 ± 115
Possible sepsis	31	66 ± 13	63	14 ± 6	209 ± 100	114 ± 103
N-I SIRS	39	59 ± 19	79	13 ± 6	197 ± 97	67 ± 79
No SIRS	14	54 ± 15	41	8 ± 3	184 ± 82	42 ± 45
Control	20	37 ± 11	52	8 ± 4	292 ± 47	<5

^a^CRP, C-reactive protein; N-I SIRS,= Non-infectious systemic inflammatory response syndrome; WBC, White blood cell. Values are presented as means ± standard deviation.

**Table 2:** **Site of isolation and organisms identified in patients with definite sepsis or possible sepsis** ^**a**^

**Site (number of patients)**		**Organism (number of patients)**
Definite sepsis		
	Lung (BAL/tracheal aspirate) (*n* = 20)	*Escherichia coli* (*n* = 4), *Enterobacter* (*n* = 2), *Haemophilus influenzae* (*n* = 1), *Klebsiella pneumoniae* (*n* = 1), *Pseudomonas aeruginosa* (*n* =8), *Serratia marcescens* (*n* = 2), *Staphylococcus aureus* (*n* = 2)
	Blood (*n* = 11)	Coagulase –ve *Staphylococcus* (*n* = 5), *Enterococcus faecalis* (*n* = 1), *Escherichia coli* (*n* = 1), *Morganella marganii* (*n* = 1), *Pseudomonas aeruginosa* (*n* = 2), *Staphylococcus aureus* (*n* = 1)
	Drain fluid (pleural/ascitic) (*n* = 3)	*Klebsiella pneumonae* (*n* = 1), *Serratia marcessans* (*n* = 1), *Streptococcus species* (n = 1)
	Line tip (*n* = 5)	*Enterococcus species* (*n* = 1), *Escherichia coli* (*n* = 2), *Klebsiella pneumoniae* (*n* = 1), *Pseudomonas aeruginosa* (*n* = 1)
	Catheter/urine (*n* = 4)	*Escherichia coli* (*n* = 2), *Klebsiella pneumoniae* (*n* = 2)
	Throat/rectal screen (*n* = 8)	*Escherichia coli* (*n* = 5), *Klebsiella pneumoniae* (*n* = 1), *Morganella marganii* (*n* = 1), *Salmonella species* (*n* = 1)
Possible sepsis		
	Lung (*n* = 2)	*Enterobacter* (*n* = 1), Methicillin-resistant *Staphylococcus aureus* (*n* = 1)
	Line tip (*n* = 4)	Coagulase-ve *Staphylococcus* (*n* = 2), *Escherichia coli* (n = 2)
	Catheter/urine (*n* = 3)	*Escherichia coli* (*n* = 1), *Klebsiella pneumoniae* (*n* = 1), *Pseudomonas aeruginosa* (*n* = 1)
	Throat/rectal screen (*n* = 3)	*Pseudomonas aeruginosa* (*n* = 1), *Escherichia coli* (*n* = 1), *Citrobacter freundii* (*n* = 1)
	No organism identified (*n* = 20)	

^a^BAL, Bronchoalveolar lavage; CRP, C-reactive protein; N-I SIRS, Non-infectious systemic inflammatory response syndrome. Values are presented as means ± standard deviation.

### Identification of immature neutrophils

**Figure 1:**
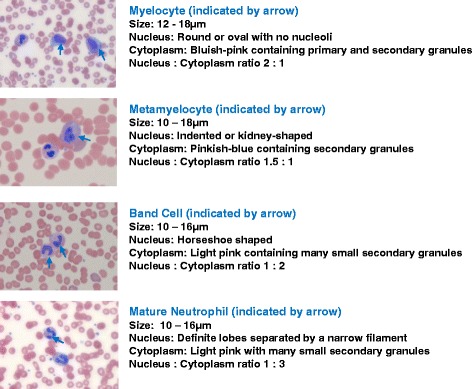
**Morphological features of myelocytes, metamyelocytes, band cells and mature neutrophils stained with Wright-Giemsa stain on blood smears.**

Although automated procedures are available to identify immature neutrophils in blood samples, none are able to accurately discriminate band cells, myelocytes and metamyelocytes from one another. Thus, morphological and staining characteristics remain the gold standard for the identification of myeloid progenitors. Blood smears were prepared from peripheral blood samples (4.5 ml), anticoagulated with ethylenediaminetetraacetic acid (K_3_EDTA; BD Biosciences, Oxford, UK) and stained with Wright-Giemsa stain (Sigma-Aldrich, Poole, UK). To determine the distribution of immature neutrophils, 200 cells of the granulocyte lineage were examined in the smears (×40 magnification) by an experienced haematologist, who, at the time of analysis, was not provided with any patient details. Cells were identified by their morphological criteria, as illustrated in Figure 1 [4]. Briefly, myelocytes and metamyelocytes are large cells, with the former having a round or oval nucleus in a cytoplasm that is predominantly blue and the latter possessing a kidney-shaped nucleus with a pink cytoplasm. Band cells are smaller, with a characteristic horseshoe-shaped nucleus of uniform thickness; this contrasts with mature neutrophils, which have two to five distinct lobes of the nucleus separated by narrow filamentous bridges.

### Statistical analysis

Results are presented as either the median or the mean ± standard error of the mean. Differences between groups of normally distributed populations were assessed by analysis of variance and the Bonferroni *post hoc* test for multiple comparisons. The data in Table 3 were analysed by χ^2^ and Fisher’s exact tests. The receiver operating characteristic curve (ROC) was utilised to determine diagnostic accuracy, optimal cutoff values, areas under the curve (AUC) and sensitivity and specificity. Covariance between surface molecule expression and clinical variables was determined and tested for significance by linear regression and the Pearson’s product-moment correlation coefficient. Differences between populations with a non-parametric distribution were assessed by performing the Kruskal-Wallis test with Dunn’s *post hoc* test for multiple comparisons. All statistical analyses were performed using GraphPad Prism v5.01 software (GraphPad Software, La Jolla, CA, USA).

**Table 3:** **Levels of circulating immature neutrophils are elevated in systemic inflammatory response syndrome and further increased in sepsis according to the criterion of Bone** ***et al.*** **, but not the criterion of Levy** ***et al*** **.** ^**a**^

				**Percentage of subjects**		
			**Number of patients**	**Total immature cells**	**Band cells**	**Metamyelocytes + myelocytes**
**Criterion of Bone** ***et al.*** **[** 2 **]**						
	SIRS		122	66*	66	12
	No SIRS		14	29*	29	0
	Normal		20	0	0	0
**SIRS subgroups**						
		Definite sepsis	51	82**^†^	82	14
		Possible sepsis	32	63^†‡^	56	19
		N-I SIRS	39	39**^‡^	39	5
**Criterion of Levy** ***et al*** **. [** 3 **]**						
	SIRS		122	22	22	3
	No SIRS		14	21	21	0
	Normal		20	0	0	0
**SIRS subgroups**						
		Definite sepsis	51	25	25	4
		Possible sepsis	32	22	22	3
		N-I SIRS	39	18	18	3

^a^N-I SIRS, Non-infectious systemic inflammatory response. Subjects were defined as having immature neutrophils when these cells were either >10% of all neutrophils (Bone *et al.* [2]) or >10% of all neutrophils with a normal white blood cell count (Levy *et al*. [3]). Results are expressed as the percentages of subjects with total immature cells, band cells only and myelocytes and metamyelocytes only. For the Bone *et al.* and the Levy *et al.* criteria comparisons were undertaken between patients with SIRS or no SIRS and normal subjects. Patients with SIRS were further subdivided into categories of definite sepsis, possible sepsis and N-I SIRS. *P <0.01; **P <0.001; ^†^ *P* <0.05; ^‡^ *P* <0.05 (χ^2^ analysis and Fisher’s exact test).

## Results

### Distribution of immature neutrophils in the blood of patients with systemic inflammatory response syndrome

The prevalence of immature neutrophils in the circulation of 136 patients admitted to the ICU is presented in two formats: the first complies with the proposal of Bone *et al.* [2], in which immature neutrophils (band cells) comprise >10% of the granulocyte population, and the second is in accord with the recommendation of Levy *et al.* [3], which also states that immature neutrophils constitute >10% of blood granulocytes, but in patients with normal WBC counts.

Table 3 shows that, with the Bone *et al.* criterion, the incidence of immature neutrophils was increased in patients with SIRS (mean = 66%) compared with critically ill patients with no evidence of SIRS (mean = 29%; *P* <0.01) and with healthy control subjects (0%). The increase in immature cells was due to the increased levels of band cells. The patients with SIRS were further categorized into those with definite sepsis, possible sepsis and N-I SIRS (see Material and Methods). The prevalence of band cells was higher in patients with definite sepsis (mean = 82%) than in the group with possible sepsis (mean = 63%; *P* <0.05) and in the N-I SIRS group (mean = 39%; *P* <0.001). The incidence of band cells in patients with N-I SIRS did not differ from that of patients with no SIRS. These findings suggest that the entry of band cells into the circulation may be a particular feature of sepsis. Myelocytes and metamyelocytes were present in a small number of patients with SIRS, but including these cells in the analysis did not modify the results.

When we applied the Levy *et al.* criterion to our results, only a minority of patients with SIRS (mean = 22%) were found to have elevated immature cells in the circulation (Table 3). Similar findings were also obtained in patients with definite or possible sepsis, patients with N-I SIRS and patients with no SIRS. Thus, restricting the monitoring of immature neutrophils to patients with normal WBC counts did not differentiate any of the patient groups.

**Figure 2:**
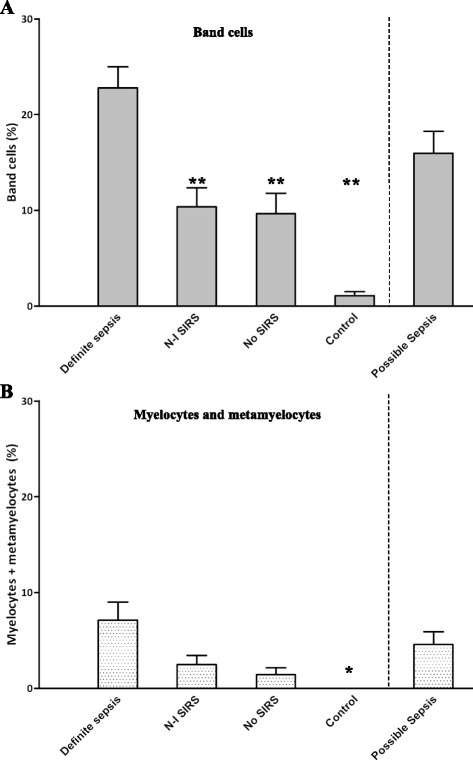
**Increased prevalence of immature neutrophils in patients with definite sepsis.** The results are expressed as the mean percentage of **(A)** band cells and **(B)** myelocytes and metamyelocytes in the blood of patients with definite sepsis (*n* = 51), non-infectious systemic inflammatory syndrome (N-I SIRS) (*n* = 39), no SIRS (*n* = 17), healthy control subjects (*n* = 14) and patients with possible sepsis (*n* = 32). Vertical bars denote standard error of the mean. Differences between the groups were assessed by analysis of variance with the Bonferroni post-test for multiple comparisons. **P <0.01 compared with definite sepsis; *P <0.05 compared with definite sepsis.

**Figure 3:**
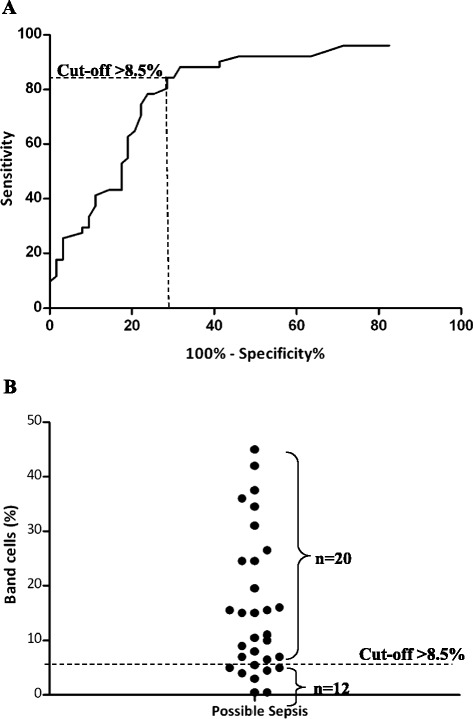
**Receiver operating characteristic curves of the percentage of band cells to discriminate definite sepsis from non-infectious systemic inflammatory response syndrome, no systemic inflammatory response syndrome and healthy controls. (A)** For identifying patients with definite sepsis, the optimal cutoff value of >8.5% band cells returned a sensitivity of 84.3% (95% confidence interval (CI) = 71.4% to 92.9%), a specificity of 71.4% (95% CI = 58.7% to 82.1%) and a likelihood ratio of 13.9. The area under the receiver operating characteristic curve was 0.80 (95% CI = 0.72 to 0.88). **(B)** On the basis of the optimal cutoff value (>8.5%), 20 of 32 patients with possible sepsis had elevated band cells.

We next examined the total percentage of band cells and of myelocytes and metamyelocytes in the different groups of subjects, irrespective of the WBC counts. Figure 2A shows that there was a higher percentage of band cells in patients with definite sepsis (mean = 23 ± 16%; *P* <0.001) compared with those with N-I SIRS (11 ± 12%), patients without SIRS (7 ± 8%) and healthy controls (1 ± 2%). The prevalence of band cells in patients with possible sepsis was similar to that in the other patient groups. Levels of myelocytes and metamyelocytes were higher in patients with definite sepsis than in healthy controls (mean = 7 ± 14% versus 0%), but they did not differentiate any of the patient groups (Figure 2B). ROC analysis demonstrated that band cells had a sensitivity of 84% and a specificity of 71% for the detection of definite sepsis, with an optimum cutoff point of 8.5% (Figure 3). Of the patients with possible sepsis (mean =16% band cells), 63% were found to have elevated band cells using the 8.5% cutoff point, raising the possibility that just over half of the patients in this group had sepsis.

### Relationships between levels of band cells and indices of infection and systemic inflammation

**Figure 4:**
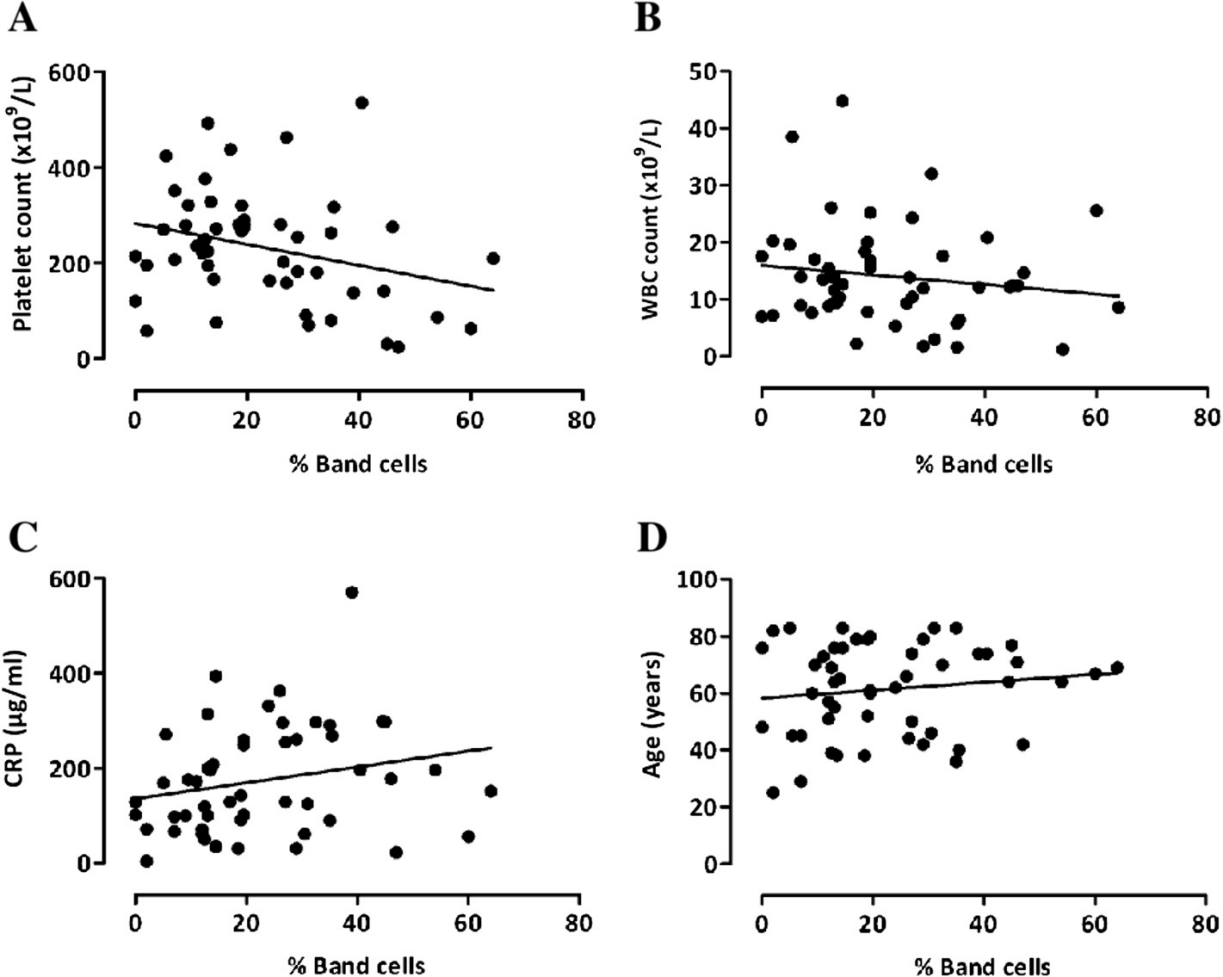
**Levels of band cells are indirectly related to the numbers of platelets in patients with definite sepsis.** Relationships were sought between the percentage of band cells and **(A)** platelet numbers, **(B)** white blood cell (WBC) count, **(C)** C-reactive protein (CRP) concentration and **(D)** patient age. Correlations were assessed by linear regression analysis and the Pearson’s correlation coefficient. The percentage of band cells was inversely related to the platelet count (*R* ^2^ = 0.08, *P = 0.04), but no other relationships were observed.

To determine if an increase in band cell distribution was associated with standard laboratory measurements of infection and inflammation, band cell levels in patients with definite sepsis were compared with the total WBC counts, platelet numbers and C-reactive protein (CRP) values. We also examined whether band cell levels were related to patient age. Figure 4 shows that the percentage of band cells was indirectly associated with the number of platelets, but not with the total WBC count, CRP concentration or patient age. Of the patients with definite sepsis, 55% had an abnormal WBC count, 23% had thrombocytopenia (that is, platelet count <150 × 10^9^/L) and 97% had a raised CRP level (>5 μg/ml). Unlike the levels of band cells, neither WBC numbers, nor platelet counts nor CRP concentrations differentiated patients with sepsis from patients with N-I SIRS.

### Association of myelocytes and metamyelocytes with mortality

**Figure 5:**
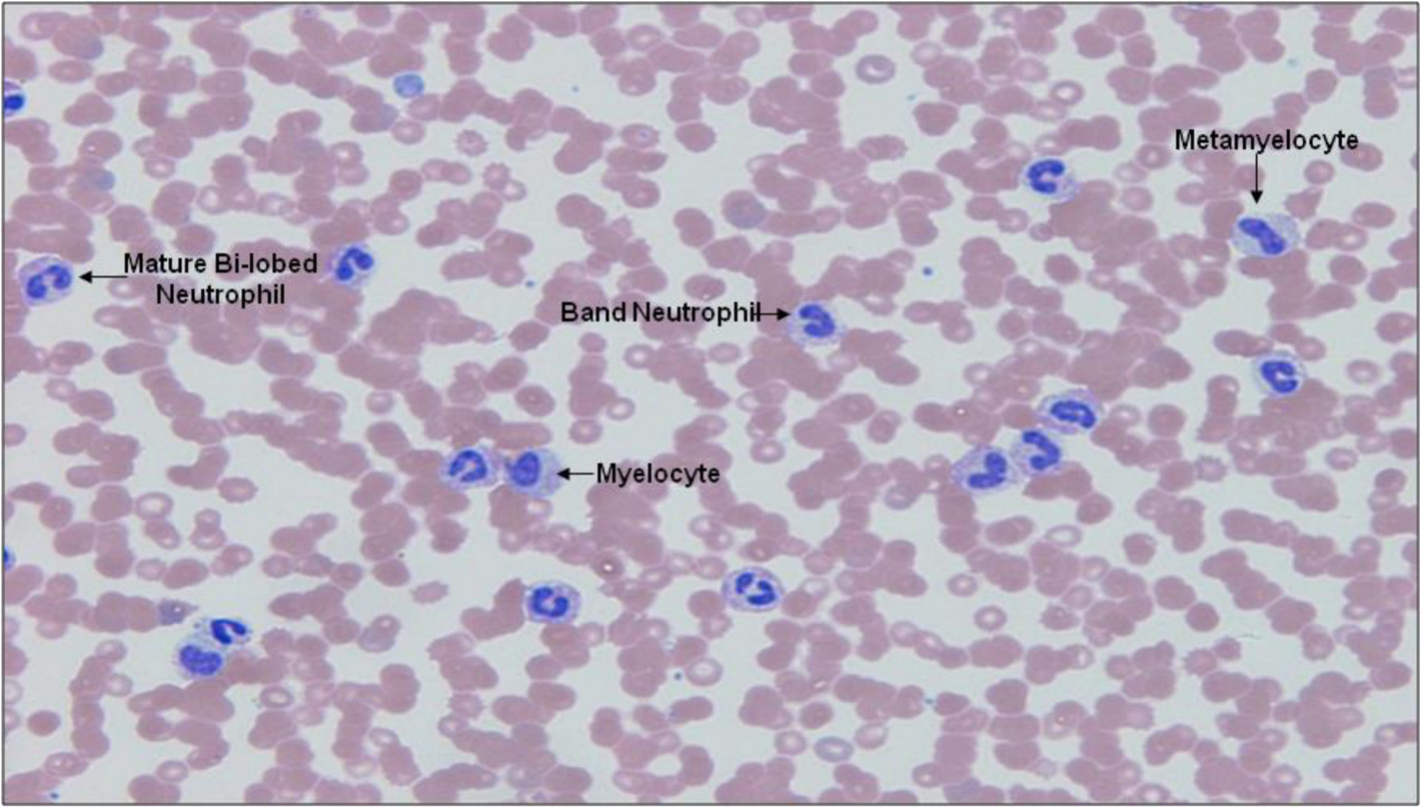
**Increased prevalence of myelocytes and metamyelocytes in the blood of a patient with sepsis who died 2 days after blood sampling.** Representative image (×100 magnification) of a whole blood smear from a patient with sepsis who died within 24 h after sampling. White blood cells were stained with Wright-Giemsa stain, allowing morphological identification of immature neutrophils. Increased numbers of myelocytes and metamyelocytes were prevalent in the blood.

**Figure 6:**
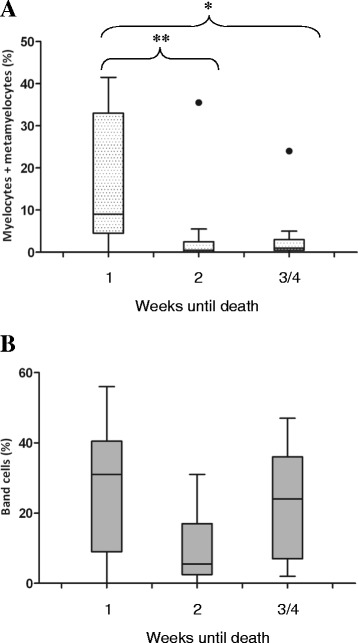
**High levels of myelocytes and metamyelocytes are associated with increased mortality.** Patients with systemic inflammatory response syndrome (*n* = 33) were categorised into three groups according to 4-week stay in the intensive care unit. These groups were deaths occurring within the first week of analysis of blood films (*n* = 14 patients), deaths occurring during week 2 (*n* = 9 patients) and deaths occurring during weeks 3 and 4 (*n* = 10 patients). The percentage of myelocytes and metamyelocytes is shown in **(A)** and the percentage of band cells in **(B)**. Data are presented as box-and-whisker plots of the median (black line), upper and lower 25% quartiles (box), range (excluding outliers) and outlier values >1.5 times the upper quartile value (black circles). Analysis was done by performing the Kruskal-Wallis with Dunn’s multiple-comparisons tests. *P <0.05 and **P <0.01 compared with patients who died within 1 week of sampling.

We also investigated whether levels of circulating neutrophils, measured at the time of patient entry into the ICU were related to outcome. Of the 136 patients investigated, 24% died within 30 days of ICU stay. Myelocytes and metamyelocytes were often present in the blood of these patients, and, in a patient who died within 24 hours of blood sample analysis, the cells comprised nearly 40% of all neutrophils (Figure 5). In Figure 6A, it is apparent that the 14 patients (9 with sepsis and 5 with N-I SIRS) who died within 1 week of blood sample provision had significantly higher levels of myelocytes and metamyelocytes (median = 9%; range = 0% to 42%) than the 9 patients who died within 2 weeks (median = 0.5%; range = 0% to 35%; *P* <0.05) and the 10 patients who died within 3 to 4 weeks (median = 1%; range = 0% to 26%; *P* <0.01). Two of the patients who died within 1 week of sampling had <3% myelocytes and metamyelocytes; their deaths were due to subarachnoid haemorrhage and pulmonary sarcoidosis, respectively. For patients whose survival was >4 weeks, the median value of myelocytes and metamyelocytes was <1%, and this concentration was significantly lower than that of patients who died within the first week of sampling (*P* <0.001). Figure 6B shows that levels of band cells were not associated with patient outcome. The results of this study imply that increased levels of myelocytes and metamyelocytes may indicate poor patient survival.

## Discussion

Identifying abnormal numbers of WBCs or increased levels of circulating immature neutrophils is one of the four established criteria for diagnosing N-I SIRS or SIRS with infection (sepsis) [2,3]. Initially, Bone and colleagues proposed that elevated levels of immature neutrophils (band cells) be defined as >10% of WBCs [2], which was later modified by Levy *et al*. to >10% immature neutrophils but with a normal WBC count [3]. In the present investigation, we noted that increased levels of circulating band cells were more prevalent in patients with sepsis than in patients with N-I SIRS, an observation that was apparent when we applied the criterion of Bone *et al*. [2], but not when we used that of Levy *et al*. [3]. Thus, with respect to WBCs and sepsis, we propose that the Bone *et al*. criterion provides more diagnostic information than that of Levy *et al*. Although the occasional appearance of myelocytes and metamyelocytes had no bearing on the differentiation of the various patient groups, an increased prevalence of these cells in the circulation was associated with poor patient outcome.

An important aspect of the present study is that an experienced haematologist, who was blinded to the clinical details of the patients, identified immature neutrophils in peripheral blood smears by their characteristic morphology. Using only one haematologist eliminates interobserver variations that may arise in cytological interpretations and in the staining methods that are important for the recognition of neutrophil progenitors and their discrimination from one another [14,22]. Differences in procedures used to identify immature neutrophils could explain why some investigators propose that the increased levels they observed in the circulation are an indication or prediction of bacterial infections [12,13,16], whereas others suggest that these levels have limited diagnostic application [8].

Our present study differs from earlier investigations in two respects. First, blood samples were obtained from unselected patients within 48 hours of entry into the ICU, and all forms of immature neutrophils were included in the assessment of neutrophil progenitors. Second, the patients were retrospectively categorized by two independent consultants into those with and those without SIRS, and they were further differentiated into those with definite sepsis, possible sepsis or N-I SIRS [21]. The important distinction between definite and possible sepsis reflects the fact that ≥30% of patients in the ICU with infections do not have positive microbiological test results [23] and that experienced clinicians may disagree on whether the patient is truly infected or merely colonised. It is therefore of interest that the prevalence of band cells, which was very high in patients with definite sepsis, was also increased in the possible sepsis group compared with patients with N-I SIRS.

Neither of the consensus definitions proposed by Bone *et al.* [2] and Levy *et al.* [3] explains the rationale for selecting >10% as the cutoff point for identifying immature neutrophils in the circulation, which probably arose from the Rochester criteria for the suspicion of neonatal sepsis [24]. However, this value of >10% approximates the optimum 8.5% cutoff point generated by our ROC analysis, which had a sensitivity of 84% and a specificity of 71% for using band cells to identify bacterial infections in patients with definite sepsis. Levels of band cells were not associated with WBC counts or with CRP concentrations, but they were indirectly related to platelet numbers. The number of WBCs and the concentration of CRP were similar in patients with definite sepsis, those with possible sepsis and those with N-I SIRS. We therefore suggest that identifying elevated levels of immature neutrophils is more helpful than abnormal total WBC counts in characterising patients with sepsis.

Because immature neutrophils, by definition, are not fully functional, their increased prevalence in the circulation of patients with sepsis might be expected to impede bacterial elimination. This may not be the case, however. The function of myeloid progenitors within the bone marrow improves during differentiation, such that the activities of band cells may be similar to those of mature neutrophils [25]. Indeed, in patients with sepsis who have both granulocytosis and increased levels of immature neutrophils, the overall phagocytic function of neutrophils is comparable to that of healthy control subjects [26].

An interesting and unexpected finding of our study was the relationship between the percentage of myelocytes and metamyelocytes and the time to patient death. Such an association was not seen with band cells, which is in agreement with an earlier observation that levels of band cells do not predict patient mortality [27]; however, it is not in accord with a report that band cells, identified by the phenotypes CD10^dim^ and CD16^dim^, predict sepsis deterioration [14]. An increase in the percentage of immature neutrophils, other than band cells, has been reported to discriminate complicated from uncomplicated sepsis [15], although researchers in another study claimed that these cells predict infection [16]. In both of these investigations, researchers employed an automated procedure to identify immature neutrophils. In the present study, we found that the prevalence of myelocytes and metamyelocytes identified by their distinct morphologies did not differentiate patients with sepsis from those with N-I SIRS. In health, myelocytes and metamyelocytes appear rarely in the circulation, but they are present in neonates with sepsis [28,29]. It is possible that, in SIRS, raised levels of myelocytes and metamyelocytes not only may serve as surrogate markers of poor outcome but also may directly participate in the initiation of organ failure. Immature neutrophils possess membranes that are more rigid than that of segmented neutrophils [30,31], and this difference in cellular rheology could account for the accumulation of immature neutrophils in the pulmonary microvasculature upon release from the bone marrow [32-34]. Prolonged interactions with vascular endothelium could initiate occlusions, hypoxia and hypoperfusion, and, in children with Gram-negative bacteraemia, elevated numbers of circulating immature neutrophils could be inducing microvascular obstruction [35]. In patients with septic shock, it was recently shown that those patients whose neutrophil numbers were below the upper limit of normal values died earlier than those with higher numbers of neutrophils [20]. We found no association between the number of neutrophils and the time to patient death. However, it is conceivable that the early onset of death in the septic shock study was influenced by the increased prevalence of myelocytes and metamyelocytes, but their distribution in the circulation was not investigated.

In relation to the association of myelocytes and metamyelocytes with patient outcome, a limiting aspect of the present study is that analysis of blood films was undertaken only during the first 48 hours of patient entry into the ICU. Most deaths occur at a later stage of ICU stay; therefore, sequential sampling of patient blood samples might have proven to be more informative. Accordingly, a study is in progress to regularly monitor the distribution of myelocytes and metamyelocytes in patients confined to the ICU for up to 28 days. Another limitation of the present investigation was that the age group of the healthy control subjects was younger than that of patients with SIRS. Although we are unaware of reports stating that immature neutrophils are present in the circulation of healthy elderly subjects, there were no differences in age between the patients in our various subgroups (that is, sepsis, possible sepsis and N-I SIRS).

To date, few researchers have used immature neutrophils as a test for the characterisation of patients with SIRS and/or sepsis, possibly because of the expertise needed for the morphological identification of these cells by manual microscopy, which is time-consuming and labour-intensive, and possibly because three of the SIRS criteria (temperature <36°C or >38°C, heart rate >90 beats/min and respiratory rate >20 breaths/min) are easily obtainable at the bedside. We believe that in order for the monitoring of immature neutrophils is to become more widespread, the cells will need to be identified by automated procedures. The current use of commercial analysers is constrained by their inability to recognise all forms of immature neutrophils. For example, the Sysmex XE-2100 (Sysmex Corporation, Kobe, Japan) and ADVIA (Siemens Healthcare, Tarrytown, NY, USA) analysers recognise promyelocytes, myelocytes and metamyelocytes as immature neutrophils but not band cells [16,36-38]. Whereas neutrophils expressing CD10^dim^/CD16^dim^ are considered to be band cells [14], as far as we are aware comparable markers for myelocytes and metamyelocytes are currently unavailable. However, it is likely that this limitation will be overcome in the near future by a greater characterisation of the phenotype of myeloid progenitors [39]. The availability of smaller, less complex and cheaper analysers would provide the impetus for extensive diagnostic and prognostic studies of circulating immature neutrophils in patients with sepsis.

## Conclusions

By using the criterion of Bone *et al.* and not that of Levy *et al*., we demonstrated that increased levels of circulating band cells were more prevalent in patients with sepsis than in patients with N-I SIRS. Blood levels of myelocytes and metamyelocytes were elevated in patients who died within the first week of ICU stay, and their appearance in the circulation may be associated with increased mortality. We propose that measuring levels of immature neutrophils will improve patient management in the ICU and that major benefits will probably materialize from use of this measurement in combination with other biomarkers to provide an algorithm of improved sensitivity and specificity for the diagnosis and prognosis of sepsis.

## Key messages

- Increased prevalence of band cells in the circulation is mainly a feature of sepsis rather than N-I SIRS.
- High blood levels of myelocytes and metamyelocytes may be indicative of poor patient outcome.

## Abbreviations

BAL: Bronchoalveolar lavage
CI: Confidence interval
CIS: Clinical Information System
CRP: C-reactive protein
ICU: Intensive care unit
N-I SIRS: Non-infectious systemic inflammatory response syndrome
ROC: Receiver operating characteristic curve
SIRS: Systemic inflammatory response syndrome
WBC: White blood cell
